# A novel subunit vaccine based on the viral protein 2 of porcine parvovirus: safety profile in bred pigs at different stages of the reproduction cycle and in offspring

**DOI:** 10.1016/j.heliyon.2019.e02593

**Published:** 2019-11-14

**Authors:** Beatriz Garcia-Morante, Marta Noguera, Sonja Klocke, Kathrin Sommer, Troy Kaiser, Verena Haist, Holger Schmidt, Philip Bridger

**Affiliations:** a Centcinc Coworking, C/, Montserrat de Casanovas 105, 08032, Barcelona, Spain; b Boehringer Ingelheim Veterinary, Research Center GmbH & Co. KG, Bemeroder Straβe 31, 30559, Hannover, Germany; c Boehringer Ingelheim Vetmedica Inc., 2621 North Belt Highway, 64506, St. Joseph, MO, USA; d BioMedVet Research GmbH, Südkampen 31, 29664, Walsrode, Germany

**Keywords:** Microbiology, Virology, Animal science, Veterinary medicine, Infectious disease, Vaccines, Vaccine safety, Mass vaccination, Porcine parvovirus 1, Pigs, Subunit vaccine

## Abstract

Porcine parvovirus 1 (PPV1) viral protein (VP) 2 is the primary antigen responsible for inducing specific protective immunity, so it is a desirable target for development of recombinant subunit vaccines to prevent PPV1 disease. The objective of this study was to evaluate repeated doses of a novel VP2-based PPV1 subunit vaccine, namely ReproCyc® ParvoFLEX, for safety in bred pigs and in offspring under experimental settings. Therefore, the investigation of safety at all breeding stages was evaluated in four independent studies involving: pre-breeding gilts (study A), breeding-age gilts and boars (study B), early and late gestating sows and offspring (study C) and lactating sows and offspring (study D). In all four studies, animals were free from PPV1 based on serology and PCR prior to inclusion. All studies comprised one or two vaccinated groups that received the PPV1 subunit vaccine and a negative control group. Thus, safety was established due to the lack of significant differences between the vaccinated groups and the corresponding unvaccinated (negative control) groups. Gilts, sows and boars were evaluated for local and systemic reactions after vaccination as well as for reproductive performance. The survival rate and average daily weight gain (ADWG) from birth to weaning in offspring was evaluated in studies C and D. Additionally, serology was determined in studies A, C and D. The vaccine was shown to be safe with no relevant significant differences between vaccinated and unvaccinated groups in any experiment. Therefore, repeated doses of ReproCyc® ParvoFLEX were safe in target animals at different stages of the reproductive cycle and in offspring, placing this vaccine as a suitable candidate for mass vaccination programs in breeding herds.

## 1 Introduction

Porcine parvovirus 1 (PPV1), recently designated as *Ungulate parvovirus* 1 [1], is one of the most important causes of reproductive failure in pigs worldwide and has implied serious economic losses in swine industries [2, 3]. Infection with PPV1 causes stillbirth, mummification of the fetus, embryonic death and infertility (termed SMEDI syndrome) and delayed return of estrus [2, 4]. Besides the virulence of the virus strain, the severity of the reproductive failure depends on the stage of gestation; PPV1 infection during the first half of pregnancy can lead to reproductive failure, whereas immunocompetent fetuses infected after day 70 of gestation usually survive the infection [5, 6, 7]. PPV1 is a small, non-enveloped virus with a single stranded DNA genome structure. It is composed of three structural proteins: viral protein (VP) 1, VP2 and VP3 [8]. VP2 incorporates the major antigenic domains and induces neutralizing antibodies. Thus, the VP2 is generally considered the key protective antigen of PPV1 vaccines [9].

Current commercially manufactured PPV1 vaccines consist of inactivated whole-virus particles adjuvanted with oil or aluminum hydroxide [2]. Although all licensed, these vaccines are based on PPV1 strains and technologies developed more than four decades ago [10]. Furthermore, the production processes of the available PPV1 vaccines are expensive, laborious, risky as well as animal consuming because of the necessity to grow and handle copious quantities of infectious virus using primary explanted cell cultures [11]. Altogether, these reasons justify the development of alternative vaccines.

Indeed, PPV1 modified live virus, subunit and live-vectored vaccines have been described and tested under experimental settings [11, 12, 13, 14, 15, 16], though none of them have been successfully licensed. ReproCyc® ParvoFLEX (Boehringer Ingelheim Vetmedica GmbH, Ingelheim am Rhein, Germany) is a recently licensed monovalent subunit vaccine for pigs based on a recombinant baculovirus expression system producing the protective VP2 protein of PPV1. This innovative vaccine is adjuvanted with the same proprietary water-based polymer (Carbopol®) as contained in ImpranFLEX® (Boehringer Ingelheim Vetmedica GmbH, Ingelheim am Rhein, Germany). Carbopol® has recently been shown to elicit robust humoral and cellular immunity to some vaccine formulations in pigs [17, 18].

Demonstrating the safety of subunit vaccines in the target species is crucial for regulatory approval and public acceptance. Therefore, four studies were designed to address the safety requirements for veterinary vaccines in the European Union (EU). All the studies were conducted according to the OECD Principles of Good Laboratory Practice (ENV/MC/CHEM 98), the EU Directive 2001/82/EC, as amended by the Directive 2009/9/EC, and the *European Pharmacopoeia* (Ph. Eur.): 5.2.6. Evaluation of safety of veterinary vaccines and immunosera (04:2013) and 01/2017:0965, Porcine Parvovirosis Vaccine Inactivated. The principal aims of these studies were to demonstrate that the aforesaid PPV1 subunit vaccine is safe in all stages of the reproductive cycle: pre-breeding gilts (study A), breeding-age gilts and boars (study B), early and late gestating sows and offspring (study C) and lactating sows and offspring (study D). Safety was assessed by comparison of the clinical observations of general health, body temperature, local injection reactions and reproductive performance in bred pigs as well as by survival rate and average daily weight gain (ADWG) from birth to weaning in the offspring to a reference (negative control) group.

## 2 Materials and methods

### 2.1 Studies characteristics and design

Four blinded and randomized studies were conducted at separate times and in different facilities. Studies A and D were performed at Boehringer Ingelheim Veterinary Research Center (BIVRC) GmbH & Co. KG (Hannover, Germany), study B was done at Boehringer Ingelheim Vetmedica, Inc. (BIVI)-Sioux Veterinary Research Center (Sioux Center, IA, USA) and study C was conducted at BioMedVet Research GmbH (Walsrode, Germany). All studies accomplished in Germany were carried out under approval of the Ethical Committee of the LAVES organization (Lower Saxony State Office for Consumer Protection and Food Safety) whereas the study carried out in the USA was approved by BIVI Institutional Animal Care and Use Committee (IACUC). In studies A and B, water and feed (without therapeutic antimicrobials) were available *ad libitum* whereas the sows from studies C and D were fed on the basis of the observation of their appetite and nutritional requirements with commercially available complete diets for pregnant or lactating pigs, which did nominally not contain any therapeutic antibiotics. Animals were commercially sourced German Landrace or crossbreed; they were not vaccinated against PPV1 prior to inclusion and were free from PPV1 based on serology and PCR. They were allowed to acclimate for a minimum of 6 days prior to the start of each study (i.e. first day of treatment, study day [SD] 0) and, at the end of the animal phase, all animals were euthanized and subjected to necropsy examination. All studies (detailed further below) included one or two test groups that received the PPV1 subunit vaccine and a negative control group.

#### 2.1.1 Study A (pre-breeding gilts)

Sixteen-week-old gilts were allocated into two comingled groups. Group Vac-A (n = 10) received 2 mL intramuscular (i.m.) of the PPV1 subunit vaccine twice, two weeks apart, on SD 0 and SD 14. On the same days, the negative control (group NC-A; n = 5) received 2 mL i.m. of sterile carbomer adjuvant (ImpranFLEX®). Necropsies of all study animals were performed on SD 28.

#### 2.1.2 Study B (breeding-age gilts and boars)

Eighteen 22- to 26-week-old gilts and boars were stratified within gender by room and randomized within rooms such that each pen consisted of three animals: two administered the PPV1 subunit vaccine and one administered phosphate-buffered saline (PBS). In total, eight gilts and four boars were vaccinated (group Vac-B; n = 12) whereas four gilts and two boars received PBS (group NC-B; n = 6). A 2-mL dose of each treatment was administered i.m. on SD 0 and SD 14 and all animals were euthanized on SD 28.

#### 2.1.3 Study C (early and late gestating sows and offspring)

This study was conducted in two separate phases in which twenty-five 7- to 8-month-old pregnant primiparous sows were used. In phase 1, fifteen sows were randomly allocated into two groups for safety evaluation when vaccinated 3 times at the beginning of gestation. Thus, sows received an i.m. administration of 2 mL of the PPV1 subunit vaccine (group Vac_1_-C; n = 10) or of NaCl solution (group NC-C; n = 5) on SD 0, 21 and 35, which corresponded to day 43, 64 and 78 of gestation, respectively. In phase 2, the remaining ten pregnant sows were allocated to group Vac_2_-C that served to evaluate safety at the end of gestation. Then, sows received a 2-mL i.m. administration of the PPV1 subunit vaccine on SD 0, 21 and 35 that corresponded to day 71, 92 and 106 of gestation, respectively. The group NC-C served as negative control for both study phases. After farrowing, piglets were included in the study on their first day of life. The animal phase was ended at weaning (21 days after parturition), when all sows were euthanized.

#### 2.1.4 Study D (lactating sows and offspring)

Thirteen sows in the late stage of gestation were included in the study and randomly assigned to two groups. First vaccination took place two days after the last sow had farrowed (SD 0). The vaccinated group (group Vac-D; n = 9) received 2 mL i.m. of the PPV1 subunit vaccine twice within a 2-week interval (SD 0 and SD 14). The negative control (group NC-D; n = 4) received isotonic NaCl solution twice within the same 2-week interval. After farrowing, piglets were included in the study on their first day of life. The animal phase ended on SD 28, when all the sows were euthanized and necropsied.

### 2.2 Vaccination

All treatments were administered i.m. into the neck musculature caudal of the ear base. On SD 0, the PPV1 subunit vaccine or the corresponding negative control product was administered in the right neck while treatments on SD 14 were administered in the left side of the neck. In the study C, animals were treated three times; into the left (on SD 0 and 35) or right (on SD 21) side of the neck in each of the two phases. All injections were given at a volume of 2 mL per animal, and, as blinded studies, all vaccinations were performed by an individual not involved with data collection. In all studies, the vaccine was prepared at BIVI (St. Joseph, MO, USA); all batches produced with this purpose were formulated with 10 μg of VP2-protein of the PPV1-27a isolate [19] per 2-mL dose, which correlates with the maximum antigen content for this vaccine.

### 2.3 Criteria for safety assessment

**Fig. 1:**
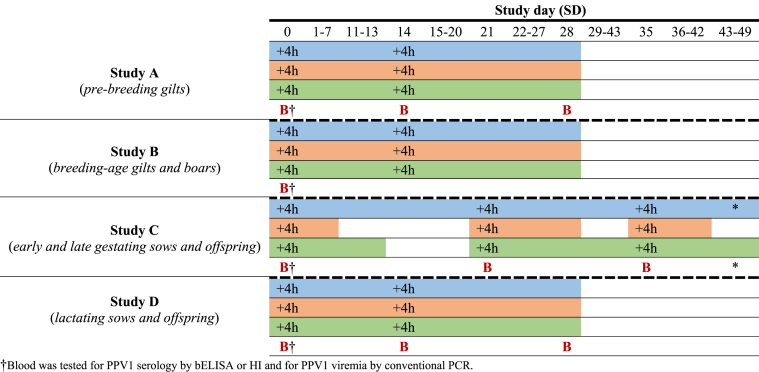
Time points and time periods of assessment of clinical observations (blue), temperatures (orange) and injection site observations (green) as well as blood sampling points (B) in gilts or sows from studies A, B, C and D. *In the study C, clinical observations lasted until farrowing, when the last blood sample was obtained.

Time points and time periods of safety parameters assessment as well as blood sampling time points in animals within each of the four studies are represented in Fig. 1. Clinical observations, body temperatures and injection site observations were recorded at the same time points and consecutively across studies A, B and D. Thus, animals were examined daily from SD 0 until necropsies (SD 28); on treatment days (SD 0 and SD 14), examinations were performed prior to and 4 h after administration of the products. Besides, clinical observations were conducted from SD 0 to farrowing (≈SD 71) in study C, though body temperature measurements and injection site observations were made prior to and 4 h after treatment on SD 0, 21 and 35 in each phase. Then, daily temperature measurements and examinations of injection sites were continued up to 7 or 14 days after the corresponding treatment, respectively.

#### 2.3.1 Clinical observations of general health

Clinical signs of disease were assessed as formerly described for breeding animals [20]. The scored categories included “behavior”, “respiration”, “digestion” and “other”. However, another category was added in studies C and D for gestating and lactating sows, which included vaginal discharge record (i.e. no discharge, mucous, purulent or bloody discharge).

#### 2.3.2 Body temperatures

Rectal temperatures were measured using self-calibrated digital thermometers in studies A, C and D. Rectal temperatures were taken first when handling the animals and were recorded in degrees Celsius units (°C). In the study B, body temperature measurements were performed by means of implantable programmable temperature transponders (IPTT-300, BioMedic Data Systems [BMDS], Inc., Seaford, DE, USA). The transponders were implanted i.m. in the trapezius muscle of the pigs before the start of the study and body temperatures were collected at approximately the same time each day using the BMDS Smart Probe scanner (Model DAS 7007R) and recorded in °C.

In studies A, C and D, the mean rectal temperature per animal measured before the start of each study (pre-treatment) was used as a covariate for all post-treatment time points. Then, pyrexia was defined as an increase in temperature of ≥1.5 °C respective to the baseline temperature of the individual animal. Absolute and relative frequencies of animals with pyrexia were calculated. In study B, least squares mean (LSM) of body temperatures by group, day and sex (gilts and boars) were calculated.

#### 2.3.3 Injection site observations

All injection sites were examined for redness, swelling, heat and pain as previously outlined in Piontkowski *et al.* (2016). In studies A, B and D, injection sites on the right side of the neck were monitored for signs of local reactions from SD 0 to SD 14, while the left side of the neck was examined from SD 14 onwards. Following the vaccination scheme, in the study C, injection site observations were done from SD 0 to SD 14 and from SD 35 onwards in the left neck, and from SD 21 to SD 35 in the right side of the neck.

#### 2.3.4 Reproductive performance

Reproductive performance was only assessed in the studies C and D. Around the estimated day of parturition, the sows were closely observed for any sign of dystocia. As soon as possible after birth (i.e. within 24 h), the total litter size was determined, and piglets were examined for external abnormalities. A live piglet at birth was defined as any piglet that was healthy, weak or crushed (dead after breathing). At the end of each study, piglets were weaned (21 or 28 days of life in study C and D, respectively) and the number of weaned piglets (piglets that completed the study) noted.

#### 2.3.5 Piglet average daily weight gain

This parameter was only assessed in the studies C and D. Individual body weights (kg) of all piglets were collected on the day of birth and then at weaning or on the day a piglet was found dead. ADWG was determined for all surviving piglets.

### 2.4 Blood collection for viremia and serology

Blood was collected by jugular venipuncture from all animals in all studies and processed for serum, which was aliquoted into appropriate tubes and held at either 2 °C–8 °C or -20 °C (±5 °C) before testing. In all studies, blood was obtained prior to or on the day of first vaccination (SD 0; Fig. 1). These samples were tested for the presence of PPV1-specific antibodies and for PPV1 viremia by means of PCR. Then, blood sampling was done on SD 14 and SD 28 in studies A and D and on SD 21, SD 35 and on the farrowing day in study C. In studies A, C and D, serum was analyzed by blocking enzyme-linked immunosorbent assay (bELISA; INgezim® PPV Compac, INGENASA, Spain). In all instances, the performance of the test was self-verified, and it was used and interpreted according to the manufacturer's recommendations. Singularly, serum samples collected in study C were sent to the Veterinary Diagnostic Laboratory (VDL; Iowa State University, Ames, IA, USA) and externally tested by hemagglutination inhibition (HI) assay for serology.

### 2.5 Polymerase chain reaction method for the detection of PPV1

Porcine parvovirus 1-DNA amplification was a modification of the procedure described by Molitor *et al.* (1991). The primers applied in the PCR reaction were derived from the DNA sequences common to two isolates of PPV1: NADL-8 and NADL-2 (Molitor *et al.*, 1991). Briefly, 8 μL of the DNA preparation was used as PCR template and amplification was performed in a final volume of 50 μL. The reaction mixture consisted of 0.2 μM of each primer, 0.2 mM of each nucleotide, 1 × PCR buffer (QIAGEN, Hilden, Germany) and 2.5 U of *Taq* DNA polymerase (QIAGEN). RNase-free water (34.5 μL; QIAGEN) was added to prevent evaporation of the reaction mixture. The reaction was performed in a thermocycler under the following conditions: initial heating at 94 °C for 5 min and 38 cycles, denaturation at 94 °C for 30 s, annealing at 55 °C for 30 s, and extension at 72 °C for 45 s. Ten µL of the amplified product (158 base pairs) were collected and directly analyzed on an agarose gel by electrophoresis. The sensitivity and specificity of this technique was previously assessed by means of testing different samples (e.g. serum, thoracic washes) containing defined amounts of PPV1; the limit of detection obtained was at least 2.09 log10/mL of PPV1.

### 2.6 Postmortem examinations

At the end of the *in vivo* phase, all animals, but weaned piglets (studies C and D), were euthanized and submitted for necropsy evaluation. The whole carcass was macroscopically inspected with special focus on injection sites. In studies A, B and C, a sample of the tissues of both injection sites (skin, subcutis and underlying muscle) were formalin-fixed, embedded in paraffin, sectioned, and stained with hematoxylin and eosin stain for possible histopathologic evaluation. If evaluated, accumulations of small to medium numbers of lymphocytes, histiocytes or eosinophilic granulocytes at the injection site were considered a normal reaction to the vaccine or reference item. Microscopic injection site reactions were considered present if abnormal inflammation at the injection site was observed.

### 2.7 Statistical analysis

The statistical analyses and data summaries were done using SAS software version 9.3 or a higher version (SAS Institute Inc., Cary, NC, USA). All data were summarized descriptively based on the type of variable and analyzed assuming a completely random design structure. Tests on differences were designed as 2-sided tests at α = 0.05, with differences considered significant if *p* ≤ 0.05. Animals removed after the start of the study (SD 0) were considered for the respective parameter of analysis until date of exclusion. In the same line, litters from removed sows were fully excluded from analyses.

For the statistical analysis, each individual sow or litter was used as an experimental unit. The data were summarized in frequency tables or tables with descriptive statistics such as number of animals, minimum, maximum, median, mean with 95% confidence interval (CI) and standard deviation (STD). The main objective of the statistical analysis was the comparison of the groups vaccinated with the PPV1 subunit vaccine (i.e. groups Vac-A, Vac-B, Vac_1_-C, Vac_2_-C and Vac-D) with the corresponding unvaccinated (negative control) groups (i.e. NC-A, NC-B, NC-C and NC-D). Explicitly, generated frequency tables for clinical observations, pyrexia, injection site reactions and seropositive animals per time point of investigation were analyzed using Fisher's exact test. For differences in temperatures at post-vaccination time points, an analysis of variance and subsequent t-test using baseline as a covariate was carried out in studies A, C and D. In study B, LSM were compared by a repeated measures ANOVA. Differences on piglet's ADWG from studies C and D were tested by analysis of variance and subsequent t-tests using also baseline as a covariate. Last of all, differences in the percentage of live piglets per sow at birth and at weaning were tested using the Wilcoxon Mann-Whitney test.

## 3 Results

### 3.1 Clinical observations

No gilt, sow or boar was found dead during the course of any of the studies. Importantly, no significant differences regarding clinical observations between the vaccinated groups and the non-vaccinated groups with respect to the frequency of animals showing at least one specific finding in the scored parameters were observed in any of the four studies (*p* > 0.05). In study D, one sow (group Vac-D) was euthanized at SD 24 due to severe loss of weight and bad general health condition. At necropsy, it presented a severe subacute necrotizing inflammation which reached from a skin ulceration proximo-lateral to the left elbow. At that level, the animal also showed focal fibrino-necrotizing pleuropneumonia.

### 3.2 Body temperatures

In studies A, C and D, no animal showed an increase in rectal temperature of ≥1.5 °C within the seven days after vaccination when compared to the baseline value taken prior to the first treatment (SD 0). With none of the study animals classified as pyretic in any of the monitoring periods, pyrexia was not statistically evaluated.

**Fig. 2:**
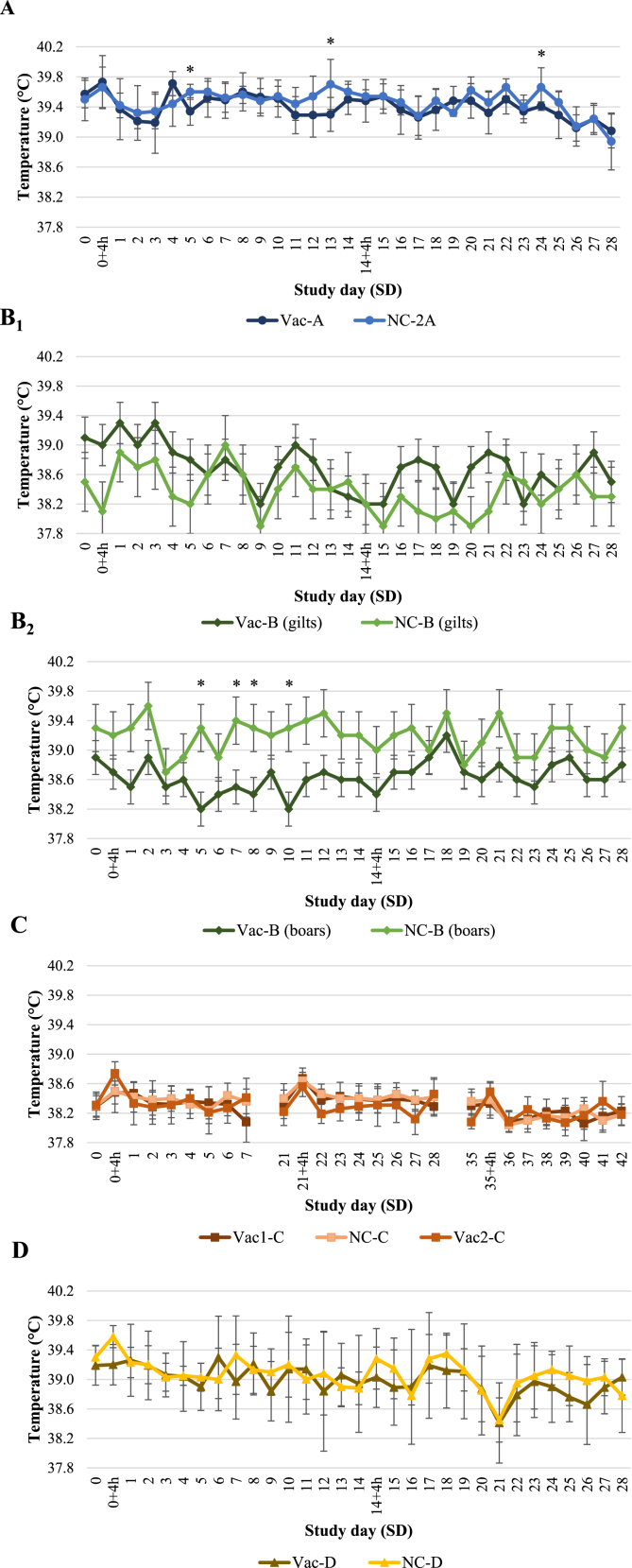
Mean rectal temperatures (±STD) from study A (A), study C (C) and study D (D) and LSM (±STD) body temperatures of gilts (B_1_) and boars (B_2_) from study B of vaccinated as well as negative control groups. *Statistical differences (*p* < 0.05) between groups. Vac = vaccinated groups; NC = negative control groups.

Mean rectal temperatures within each group from studies A, C and D as well as LSM of body temperatures from study B are shown in Fig. 2. In studies A, C and D, there were no statistically significant differences between the vaccinated groups and the negative control groups at baseline. Nonetheless, a significant difference between the vaccinated group and its non-vaccinated counterpart was found at different time points along studies A and B. In study A, the mean rectal temperature was significantly higher in the non-vaccinated animals (group NC-A) than in the vaccinated ones (group Vac-A) at SD 5 (*p* = 0.0169), 13 (*p* = 0.0116) and 24 (*p* = 0.0162). Similarly in study B, the LSM body temperature of the boars was significantly higher in the group NC-B when compared to the group Vac-B at SD 5 (*p* = 0.0071), 7 (*p* = 0.0323), 8 (*p* = 0.0376) and 10 (*p* = 0.0059).

### 3.3 Injection site observations

Reactions at the injection sites in the study A consisted in redness and swelling. Redness of the injection site was seen in two animals in group Vac-A after the first vaccination. At second vaccination, two animals of the group Vac-A and one in the group NC-A showed also redness at the inoculation site. In all cases, redness was of slight or moderate degree (score 1 or 2) and of a maximum duration of 2 days. Furthermore, minimal to slight swelling of the injection site was seen in six animals in the vaccinated group Vac-A only after the second vaccination. The size of swelling ranged from 0.1 to 0.5 cm and was only palpable at the injection site. The maximum duration of swelling was 5 days. No animal exhibited any injection site reaction post-treatment in study B. In studies C and D only redness was observed at the injection site. Slight redness (score 1) was observed in one animal 1 h after the 1^st^ vaccination (SD 0) during phase 2 (late gestation) of the study C. In study D, a total of nine animals (six from the vaccinated group Vac-D and three from the non-vaccinated group NC-D) showed mild redness at the injection sites, principally after the second vaccination. Redness disappeared 24 h after injection in all cases.

### 3.4 Reproductive performance

The proportion of live piglets at birth per litter is shown in Table 1 whereas the mean survival rate at weaning, calculated as number of weaned piglets in relation to the number of live piglets at farrowing, is exposed in Table 2 for study C and D. No statistically significant differences (*p* > 0.05) were found between compared groups nor in terms of percentage of alive pigs at birth neither in weaned pigs per litter in any of the two studies. In the study C, one sow belonging to the control group NC-C delivered stillborn piglets only, therefore, this litter was not considered for survival at weaning as well as ADWG parameters. Likewise, those piglets from the sow belonging to the group Vac-D that was euthanized on SD 24, were not considered for survival at weaning and ADWG either.

**Table 1:** Proportion (%) of alive piglets at farrowing per litter from studies C and D. No statistically significant differences (*p* > 0.05) were found between compared groups in terms of percentage of alive pigs per litter at birth.

	Group	N_total_	Min.	Max.	Median	Mean	[95% CI]	STD	*P*
Study C	Vac_1_-C	10	82	100	100	95.8	91.2, 100	6.4	0.144
	NC-C	5	0	100	93.3	74.8	22.5, 100	42.1	**---**
	Vac_2_-C	10	75	100	91.3	90.2	83.8, 100	8.9	**---**
Study D	Vac-D	9	100	100	100	100	---	---	**---**
	NC-D	4	100	100	100	100	---	---	**---**

Vac_1_-C = primiparous vaccinated early in gestation (study C).

Vac_2_-C = primiparous vaccinated late in gestation (Study C).

Vac-D = vaccinated, lactating sows (study D).

NC-C and NC-D = negative control animals (study C and D, respectively).

**Table 2:** Proportion (%) of alive piglets at weaning per litter in relation to the number of live piglets at farrowing from studies C and D. No statistically significant differences (*p* > 0.05) were found between compared groups in terms of percentage of weaned pigs per litter.

	Group	N_total_	Min.	Max.	Median	Mean	[95% CI]	STD	*P*
Study C	Vac_1_-C	10	21	100	70	59.5	36.6, 82.4	32	0.287
	NC-C	4	15	64	46.7	43.3	10.9, 75.6	20.4	**---**
	Vac_2_-C	10	56	100	82.6	79.6	69.6, 89.6	14	**---**
Study D	Vac-D	8	90	100	100	97.5	93.6, 100	4.6	0.774
	NC-D	4	93	100	96.7	96.5	90.2, 100	4	**---**

Vac_1_-C = primiparous vaccinated early in gestation (study C).

Vac_2_-C = primiparous vaccinated late in gestation (Study C).

Vac-D = vaccinated, lactating sows (study D).

NC-C and NC-D = negative control animals (study C and D, respectively).

It is worth mentioning that litters from all groups of the study C (phase 1) were found to be affected by atypical porcine pestivirus (APPV) infection. Although no abnormal histological findings were appraised in samples of the cerebrum, APPV genome was identified by PCR in the brain tissue as well as in the tonsils from piglets with clinical signs at farrowing. Such clinical signs consisted of head and/or body tremors associated with other signs namely wobbly, standing difficulties or impaired walking. In addition, a high proportion of litters (i.e. 7 out of 10 in group Vac_1_-C, all four litters from group NC-C and 6 out of 10 litters in group Vac_2_-C) had pre-terminal dead piglets, meaning animals found dead or euthanized for animal welfare reasons. Consequently, the proportion of weaned pigs was generally low in study C.

### 3.5 Piglets average daily weight gain

Piglets performance from studies C and D is summarized in Table 3. In study C, the body weights at farrowing were similar between vaccinated groups Vac_1_-C and Vac_2_-C. Considering both phases together, the ADWG did not differ significantly (*p* > 0.05) between the three groups in the 21 days period. Similarly, in study D, there was no statistically significant difference between the group Vac-D and the control group NC-D with regard to ADWG in piglets in the 28 days period considered.

**Table 3:** ADWG (kg) of piglets from farrowing to weaning at 21 and 28 days of life for study C and D, respectively. No statistically significant differences (*p* > 0.05) were found between compared groups in terms of piglets’ ADWG.

	Group	N_total_	Min.	Max.	Median	Mean	[95% CI]	STD	*P*
Study C	Vac_1_-C	64	0.10	0.35	0.27	0.25	0.24, 0.26	0.05	0.642
	NC-C	25	0.14	0.31	0.25	0.25	0.23, 0.26	0.03	**---**
	Vac_2_-C	84	0.06	0.35	0.22	0.22	0.21, 0.23	0.05	**---**
Study D	Vac-D	80	0.06	0.36	0.24	0.24	0.22, 0.25	0.06	0.780
	NC-D	49	0.12	0.33	0.24	0.23	0.22, 0.25	0.06	**---**

Vac_1_-C = primiparous vaccinated early in gestation (study C).

Vac_2_-C = primiparous vaccinated late in gestation (Study C).

Vac-D = vaccinated, lactating sows (study D).

NC-C and NC-D = negative control animals (study C and D, respectively).

### 3.6 PPV1 viremia and serological results

**Fig. 3:**
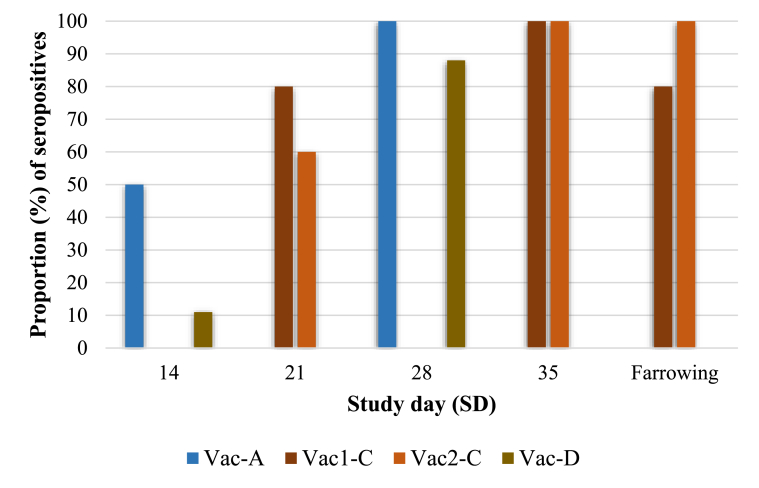
Proportion of seropositive animals against PPV1 in the vaccinated groups along the studies A, C and D. A significantly higher (*p* < 0.05) percentage of PPV1-seropositive animals to PPV1 was found from SD 21 onwards compared to the respective non-vaccinated groups (i.e. groups NC-A, NC-C and NC-D). Vac = vaccinated groups; NC = negative control groups.

All blood samples analyzed before or at the start (SD 0) of the studies were negative to PPV1 by PCR. In parallel, all study animals were negative for antibodies against PPV1 before or at the start of each of the studies. In addition, all the animals belonging to the negative control groups did not show seroconversion to PPV1 throughout any of the studies but in the study A, in which two animals showed a positive reaction in the PPV1 bELISA on SD 14. Beyond this, there was a significantly higher (*p* < 0.05) percentage of seropositive animals to PPV1 from SD 21 onwards in the vaccinated groups compared to the negative control groups in all three studies. Proportion of seropositive animals against PPV1 belonging to the vaccinated treatment groups from studies A, C and D are depicted in Fig. 3.

### 3.7 Postmortem assessment of injection sites

At the scheduled necropsies, all animals were in good body condition and none of them showed any macroscopic alterations in the area of the injection sites (i.e. left and right side of the neck), neither by visual inspection nor by palpation in any of the four studies.

In study A, where the highest proportion of injection site reactions were macroscopically observed after vaccination days, no relevant differences were found between groups Vac-A and NC-A with regard to histological findings; all 15 animals but one from the vaccinated group Vac-A, showed mild inflammatory, degenerative and/or regenerative alterations at one of both injection sites. In most cases, severity of lesions was slightly higher at the left injection site (applied on SD 14). Nevertheless, lesions were only mild to moderate and restricted to a very small area within the deeper segments of the tissue sample (i.e. skeletal muscles of the neck region). Histological findings were dominated by non-inflammatory processes such as muscle fiber degeneration, muscle fiber regeneration and fibrosis. Occasionally, (lympho-)histiocytic inflammation was observed in muscle or surrounding adipose tissue. Since no animals had macroscopic injection site reactions during the study B, no tissue samples were collected for histological evaluation. In study C, no specific (injection-related) findings were noted in either treatment site of the animals.

## 4 Discussion

Because PPV1 is prevalent in the pig population and highly stable in the environment, it gets difficult to establish and maintain breeding populations free of the virus. Consequently, a common goal in commercial herds is to maintain herd immunity against the virus by regular vaccination of breeding females [2]. While most commercial vaccines are based on chemical inactivation of tissue culture-derived virus adjuvanted with oil or aluminum hydroxide, several subunit vaccines have been described at the experimental level, with most based-on expression of the viral VP2 protein [2, 11, 13, 14]. Lately, a novel VP2 protein-based PPV1 subunit vaccine has been registered in the EU, namely ReproCyc® ParvoFLEX. This vaccine contains the same adjuvant (Carbopol®) as contained in ImpranFLEX® and as utilized consistently throughout the FLEX Family™ of Boehringer Ingelheim vaccines.

Any vaccine against PPV1 must be safe to administer, however, in that it does not cause reproductive failure, local or systemic reactions, and negatively affect piglet survivability and growth performance. Hence, a set of studies were performed to assess the safety of administration of the abovementioned novel PPV1 subunit vaccine in bred animals at different reproductive stages and in the offspring. To the authors’ knowledge, this is the first published work reporting the safety profile of a vaccine against PPV1. Remarkably, in all four studies, no PPV1-DNA and no PPV1 antibodies could be detected by PCR or bELISA in serum samples of all animals prior to the 1^st^ vaccination on SD 0. Afterwards, PPV1 antibodies could not be detected in the negative control groups except for two single animals from the study A. However, these two positive results lacked consistency as they were negative in the following sampling on SD 28 and, therefore, regarded as an unspecific reaction of the bELISA. Altogether, the conditions of these conducted studies are validated.

Injectable medicines for veterinary applications must be checked for local and systemic adverse effects during the development and registration process. In the present set of studies, no relevant differences were detected between groups for abnormal clinical findings and body temperatures. Indeed, none of the clinical observations could be attributed to the vaccine or control products administered. In study D, one sow was euthanized due to animal welfare reasons, and the necropsy findings rendered any relation to the vaccine very unlikely. In terms of body temperatures, significantly higher rectal temperatures in group NC-A (receiving placebo) than in group Vac-A (receiving vaccine) were found at three separated time points through study A. Similarly, in study B, boars from the group NC-B also had significantly higher temperatures after the first injection than the vaccinated group Vac-B. The latter was probably due to the reduced sample size as well as to the inherent variability in the measured variable, hence, the aforesaid statistical differences were considered not biologically relevant. Together, no clinically relevant differences in systemic reactions between groups could be set in any study, therefore, non-attributable to repeated doses of the PPV1 subunit vaccine.

In principle, every vaccine exhibits a different extent of local reaction at the vaccination site mainly due to the interaction with the adjuvant/s and the antigen/s [21]. Treatments performed in the present studies caused detectable local reactions within the neck tissue of the pigs in both (vaccinated and non-vaccinated) groups mostly from studies A and D; these, however, were generally small and transient. A certain degree of reactogenicity was confirmed by pathomorphological examination in study A. The microscopic appearance of the injection sites represented the typical range of lesions that can be caused by sterile intramuscular injections, particularly when administering particulate and/or inorganic substances such as adjuvanted vaccines. In any case, reactive and regenerative processes clearly outbalanced inflammatory infiltration, which can trap antigens at the injection site and prevent them from being recognized by the immune system [21]. Besides, higher severity of left side findings compared to the right side can be explained by the fact that injections into the left side of the neck took place two weeks prior necropsies, whereas injections on the right side were performed four weeks prior necropsies. Because injection sites with equal microscopic appearance occurred in animals from the negative control group, they were considered unrelated to the antigen contained in the vaccine. Moreover, the lack of reproducibility across all four studies of ongoing inflammation and of macroscopically visible alterations trigger the microscopic injection site reactions clinically negligible or irrelevant.

Porcine parvovirus 1-naive gilts and sows from studies C and D that were given repeat doses of the PPV1 subunit vaccine did not exhibit any relevant difference in the mean percentages of live piglets at birth neither at weaning compared to non-vaccinated controls. In parallel, during the suckling period, no significant differences between the vaccinated and non-vaccinated groups for the ADWG of piglets were observed. It is worth mentioning that in study C (phase 1) an APPV infection was considered to be the reason for the relatively high mortality found in piglets of both vaccinated and non-vaccinated groups. APPV has been recently identified in neonatal piglets with congenital tremors in the United States, Brazil and some countries within Europe and Asia [22, 23, 24]. Because both groups were affected in an analogous manner, this concurrent disease was not regarded to have a significant impact on the outcome of the study. Henceforth, ReproCyc® ParvoFLEX did not cause reproductive failure and negatively affect piglet survivability and growth performance.

While determinants of PPV1 virulence are mainly unknown [1, 25], many immunological studies proved that the presence of neutralizing serum antibodies in the dam is a decisive factor in the outcome of the PPV1 infection; they prevent fetal death by avoiding the virus to cross the placenta barrier [10, 25, 26]. Nonetheless, cell-mediated immunity should not be ignored since it may also play a role in controlling PPV1 reinfection [26]. In the latter respect, Carbopol® has been shown to improve cellular immunity by inducing early IFN-γ-producing cells and by preferentially driving T cell differentiation to effector phenotypes [17, 27]. In the present case, seroconversion to PPV1 vaccination was demonstrated, starting by 2-weeks after the first vaccination (from 10 to 50% of the animals). Almost all vaccinated animals became seropositive to PPV1 after the boost administration, by 4-weeks after the first vaccination. Additionally, in the study C, a third vaccine was administered at 78 (group Vac_1_-C) and 106 (group Vac_2_-C) days of gestation, becoming the 80 and 100% of the dams seropositive at farrowing, respectively. The latter, in turn, may promote a better protection in the offspring, since maternally derived antibodies are dependent on the serum and colostrum antibody levels of the dam and on the colostrum intake by the piglet [28]. The obtained results are in line with previous reported serological data, in which all sows which received an inactivated whole-PPV1 vaccine seroconverted within 5 weeks [10, 29]. These results indicate that vaccinated pigs seroconverted upon vaccination indicating exposure to the vaccine antigen.

In the present set of studies, safety was determined by evaluating systemic and local reactions to vaccination, reproductive performance at farrowing (number of live-born piglets), the number of piglets at weaning, and the ADWG. This was supported by evaluating gilts and sows for post-vaccination seroconversion to PPV1. According to these criteria, the present data demonstrated that vaccination of pigs intended for breeding with the novel VP2 protein-based PPV1 subunit vaccine adjuvanted with Carbopol® is a safe option for preventing reproductive losses associated with the PPV1 virus. Furthermore, safety at any stage of the reproduction cycle makes ReproCyc® ParvoFLEX suitable for mass vaccination protocols, which are widely implemented in the breeding herds.

## 5 Conclusion

The safety of the application of a subunit vaccine consisting of PPV1-VP2 adjuvanted with Carbopol® has been demonstrated at every time point of the reproductive stage, including all stages of gestation, pre-breeding and lactation. Hence, two or even three 2-mL doses (at the maximum allowed antigen content) of the PPV1 subunit vaccine given to animals intended for breeding resulted in no negative impact. This candidate vaccine complies with the Ph. Eur. requirements and its appropriateness for mass vaccination protocols advocate ReproCyc® ParvoFLEX as a real and competitive alternative to the classical whole cell inactivated vaccines.

## Declarations

### Author contribution statement

Beatriz Garcia-Morante: Analyzed and interpreted the data; Wrote the paper.

Marta Noguera, Kathrin Sommer, Troy Kaiser, Holger Schmidt: Conceived and designed the experiments; Performed the experiments; Analyzed and interpreted the data.

Sonja Klocke, Philip Bridger: Conceived and designed the experiments; Analyzed and interpreted the data.

Verena Haist: Analyzed and interpreted the data; Contributed reagents, materials, analysis tools or data.

### Funding statement

This work was supported by Boehringer Ingelheim Animal Health, Inc.

### Competing interest statement

The authors declare the following conflict of interests: Marta Noguera, Kathrin Sommer, Troy Kaiser, Sonja Klocke, Philip Bridger, Verena Haist; employees of Boehringer Ingelheim.

### Additional information

No additional information is available for this paper.
